# Characterization of the Complete Mitochondrial Genome of the Red Alga *Ahnfeltiopsis flabelliformis* (Rhodophyta, Gigartinales, Phyllophoraceae) and Its Phylogenetic Analysis

**DOI:** 10.3390/biology14060638

**Published:** 2025-05-30

**Authors:** Maheshkumar Prakash Patil, Jong-Oh Kim, Young-Ryun Kim, Nilesh Nirmal, Gun-Do Kim, Kyunghoi Kim

**Affiliations:** 1 Industry-University Cooperation Foundation, Pukyong National University, 45 Yongso-ro, Nam-Gu, Busan 48513, Republic of Korea; 2 Department of Microbiology, Pukyong National University, 45 Yongso-ro, Nam-Gu, Busan 48513, Republic of Korea; 3 School of Marine and Fisheries Life Science, Pukyong National University, 45 Yongso-ro, Nam-Gu, Busan 48513, Republic of Korea; 4 Marine Eco-Technology Institute, Busan 48520, Republic of Korea; 5 Department of Food Science, Institute of Nutrition, Mahidol University, Salaya 73170, Thailand; 6 Department of Ocean Engineering, Pukyong National University, 45 Yongso-ro, Nam-Gu, Busan 48513, Republic of Korea

**Keywords:** *Ahnfeltiopsis flabelliformis*, mitogenome, phylogenetic, Rhodophyta, Gigartinales, red algae

## Abstract

Red algae are recognized for their health-promoting bioactive substances and dietary fibers, making them important as functional food. In order to identify species and determine phylogenetic relationships, mitochondrial genes serve as important markers. Thus, this study sequenced the complete mitochondrial genome of *Ahnfeltiopsis flabelliformis*, compared with Phyllophoraceae species, and performs phylogenetic analysis to reveal its evolutionary position. The genome is 25,992 bp long, has 71.3% of biased AT content, and comprises 24 protein-coding genes (PCGs), 22 tRNA, and three rRNA genes. The overall base composition of its mitochondrial genome was 37.4% for A, 33.9% for T, 14.7% for G, 14.0% for C and 28.7% for GC. The gene content, annotation, and genetic makeup are identical to those of Phyllophoraceae species. Phylogenetic study based on the complete mitochondrial genome and shared mitochondrial genes revealed that the six Phyllophoraceae species form a well-supported clade. Within this clade, *A*. *flabelliformis* groups with *Gymnogongrus griffithsiae*, and together they form a distinct subclade including four species of the *Mastocarpus*. The results indicate that *A*. *flabelliformis* shares a closer evolutionary relationship with *G. griffithsiae* than with *Mastocarpus* species. Future research on *Ahnfeltiopsis* is necessary to comprehend the evolutionary history and phylogenetic relationships among species in this genus.

## Simple Summary

Red algae, particularly those in the order Gigartinales, are valuable for their bioactive compounds and hold significant economic and ecological importance. Mitochondrial genomes serve as crucial markers for species identification and phylogenetic studies. However, limited mitochondrial genome data exist for Gigartinales species. This study presents the first complete mitochondrial genome sequence of *Ahnfeltiopsis flabelliformis*, a species in the Phyllophoraceae family. The mitochondrial genome features are in line with the red algal species and phylogenetic study positions *A. flabelliformis* within a well-supported Phyllophoraceae clade. The findings of this study enhance our understanding of red algal evolutionary history and offer valuable genetic markers for future research on phylogenetics and species classification within this family.

## 1. Introduction

Rhodophyta comprises a monophyletic group of multicellular photosynthetic eukaryotes. Under the phylum Rhodophyta (comprising about 1094 genera, and 7708 species), some of the common classes of algae include Bangiophyceae (185 species), Compsopogonophyceae (70 species), Cyanidiophyceae (11 species), Florideophyceae (7155 species), Porphyridiophyceae (9 species), Rhodellophyceae (8 species), Stylonematophyceae (48 species), and Classis incertae (68 species) [1]. Of these, 968 species are placed in the order Gigartinales of the Class Florideophyceae, which is predominantly marine, containing multicellular algae. In fact, red algae are most often found in marine environments and are uncommon in freshwaters [2]. Currently, there are only a limited number of Gigartinales species (approximately 50) for which complete mitochondrial genome data is available in GenBank (https://www.ncbi.nlm.nih.gov/nuccore, accessed on 1 December 2024). So, analysis of the complete mitochondrial genome of red algae is important, which will be helpful in tracing the proper evolutionary history of red algae, comparative genomics through characteristic features, and gene transfer; it enhances phylogenetic classification and identification of species.

Red algae, particularly members of the order Gigartinales within the class Rhodophyta, are economically important and serve as rich sources of bioactive compounds with diverse biotechnological applications [3,4,5]. These include polysaccharides such as carrageenan and agar, which are used in a wide range of applications related to food, cosmetics, and medicine [6,7,8]. In recent studies, *Ahnfeltiopsis flabelliformis* has been reported for the richness of carrageenan and their biomedical applications [9,10].

*Ahnfeltiopsis flabelliformis* (Harvey) Masuda 1993 [11] is a species of red macroalgae within the family Phyllophoraceae (Phylum—Rhodophyta, Class—Florideophyceae, Order—Gigartinales), predominantly found in marine environments [1,2,12]. Three species of *Ahnfeltiopsis* (*A*. *catenata*, *A*. *flabelliformis*, *A*. *paradoxa*) have been reported in Korea, with *A. flabelliformis* being a conspicuous and common species [13,14]. Previously documented occurrences in the Far Eastern seas have been re-evaluated, with many of them being assigned to different species based on phylogenetic and morphological analysis [15]. Recent taxonomic findings highlight the significance of combining classic morphological studies with modern molecular approaches to appropriately classify as well as understand the diversity of red algae [15,16]. However, to date, there has been no complete nucleotide sequence of the mitochondrial genome of *Ahnfeltiopsis* species reported. Therefore, it is important that we perform research on complete mitochondrial genome analysis and the species’ phylogenetic position based on molecular genetics.

The objective of the present study was to analyze and annotate the complete nucleotide sequence of the mitochondrial genome of the *Ahnfeltiopsis flabelliformis*. Additionally, this research explored the phylogenetic relationship among Gigartinales species by using PCG sequences, thereby establishing a foundation for future investigations into red algae phylogenetics. Additionally, getting the full mitochondrial genomes of *A. flabelliformis* is important for finding new genetic markers that will help genetics, genetic diversity, and evolution research in the Phyllophoraceae family. This study presents the first complete annotated sequence of the mitochondrial genome for the genus *Ahnfeltiopsis*.

## 2. Materials and Methods

### 2.1. Sample Collection, Genomic DNA Extraction and Sequencing

A specimen of the red macroalga *Ahnfeltiopsis flabelliformis* (Figure S1) was captured from the shoreline of Busan, Republic of Korea (35°28′ N, 129°25′ E) in June 2023. The specimen was submitted to the Ecological Restoration Group at the Marine Eco-Technology Institute in Busan, Republic of Korea (contact: Dr. Young-Ryun Kim, yykim@marineeco.co.kr) under the voucher number PU-T01-S-MA-07. Total genomic DNA was extracted with a DNeasy Plant Kit (Qiagen, Venlo, The Netherlands) according to the manufacturer’s instructions. DNA concentration was determined by a NanoDrop spectrophotometer (Thermo Fisher Scientific D1000, Waltham, MA, USA). The genomic DNA was stored at −20 °C prior to further analyses. The *A. flabelliformis* library was prepared and sequenced by Macrogen (Daejeon, Republic of Korea; https://www.macrogen.com/ko/). The whole mitochondrial genome sequence was generated using high-throughput sequencing (Illumina HiSeq 2500 platform with insert size 350 bp and sequencing mode paired ends 2 × 150 bp).

### 2.2. Sequence Assembly, Annotation, and Analysis

Trimmomatic v0.36 was used to remove adapter sequences and low-quality reads (Q < 20) to ensure the accuracy of the analysis [17]. The mitochondrial genome was assembled by randomly sampling the cleaned reads, and only the sampled reads were used for de novo assembly. The quality of sequencing data was checked by FastQC v0.11.5 [18]. The high-quality reads were assembled into the mitochondrial genome using NOVOPlasty v4.2.1 [19]. The assembled contigs were compared with the known mitochondrial genome sequences in the NCBI database by BLAST analysis. Mitochondrial genetic code: Translation Table 4 (Mold Mitochondrial; Protozoan Mitochondrial; Coelenterate Mitochondrial; Mycoplasma; Spiroplasma) was used to annotate the mitochondrial genome using MFannot [20]. Open reading frames (ORF) finder and BLAST searches against the NCBI protein database were performed for the identification of PCGs [21]. Transfer RNAs (tRNAs) were identified using tRNAscan-SE 2.0 [22]. RNAweasel was used to confirm the location of RNAs, and to detect introns [23]. Tandem Repeats Finder program V4.09 was used for repeat sequences identification and analysis [24]. The mitochondrial genome map was produced using OGDRAW v1.3.1 [25]. The nucleotide compositions and the relative synonymous codon usage (RSCU) were determined using MEGA11 v11.2.8 [26]. Strand asymmetry was calculated in terms of formulae: GC-skew = [G − C]/[G + C], and AT-skew = [A − T]/[A + T] [27].

### 2.3. Phylogenetic Analysis

To determine the evolutionary relationship of *A. flabelliformis* within the Gigartinales species. The mitochondrial genome sequences of all available 45 red algal species were downloaded from GenBank, out of which 44 species were in-group and *Porphyra umbilicalis* (NC_018544) was selected as the outgroup (Table S1). The maximum likelihood (ML) phylogenetic analyses were performed based on complete mitochondrial genome sequences. The multiple sequences were aligned using MAFFT v7.0 online platform [28]. We used IQ-TREE’s Modelfinder to identify and select suitable models based on the Bayesian information criterion (BIC) [29] The phylogenetic tree using the maximum likelihood (ML) method was constructed in IQ-TREE, using the GTR + F + I + G4 model [30]. The generated phylogenetic trees were visualized using the iTOL v.7 web server [31].

## 3. Results and Discussion

### 3.1. Mitochondrial Genome Structure and Nucleotide Composition

**Figure 1:**
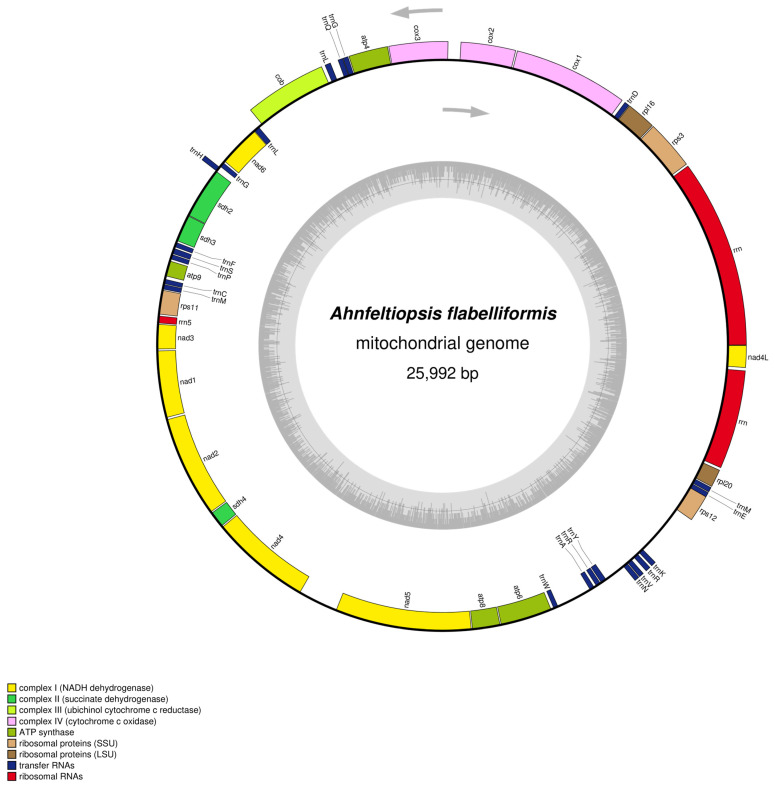
The circular mitochondrial genome of *Ahnfeltiopsis flabelliformis* (GenBank accession number: PQ685980). The arrow directions show gene orientation, the different colors reflect the groupings of functional genes together with their acronyms, and the inner circle indicates the GC content.

The *A. flabelliformis* library was made, adapter sequences were cut down, and low-quality reads were removed, and then filtered reads were subjected to de novo assembly, resulting in a contig consisting of 25,992 bp with a GC contents of 28.6% (Supplementary Table S2). Figure 1 illustrates the gene map, and Table 1 shows the characteristics of the mitochondrial genome of *A. flabelliformis*. The complete mitochondrial genome length was 25,992 bp and available in GenBank with accession number PQ685980. The mitochondrial genome of *A. flabelliformis* has 49 genes, which are made up of 24 PCGs, 22 tRNAs, and 3 rRNAs. These genes code for RNAs, respiratory chain subunits, ATP synthase subunits, ribosomal protein subunits, and independent protein translocase (Supplementary Table S3). Among the genes, 11 PCGs, 11 tRNAs, and two rRNA genes were located in the heavy (H) strand. The light (L) strand contained 13 PCGs, 11 tRNAs, and one rRNA gene (Table 1). The intergenic nucleotide analysis revealed that the junction between the *rps12* and *trnE* genes was overlapping by 16 bp, and the interval between adjacent genes was 2515 bp, which accounts for 9.67% of the complete mitochondrial genome. The complete mitochondrial genome of *A. flabelliformis* base composition was 37.4% adenine (A), 33.9% thymine (T), 14.7 guanine (G), and 14.0% cytosine (C), with a biased AT content of 71.3%. The AT content was slightly higher, and the GC content was slightly lower than the other Phyllophoraceae species (Table 2). The whole genome exhibited positive AT- and GC-skewness, suggesting a preference for using A’s over T’s and G’s over C’s. A comparison of *A. flabelliformis* with other species in the Phyllophoraceae family showed some similarities in the way their genomes were organized and the genes they contained, but there were also clear differences. The mitochondrial genomes of Phyllophoraceae species are usually 25 to 26 kb long and have 23 to 25 PCGs, 22 to 23 tRNAs, and 3 rRNAs with a biased AT composition. On the other hand, *A. flabelliformis* and *G. griffithsiae* (OP537223) share many mitochondrial genome characteristics, including nucleotide composition, biased AT composition, number of genes, and skewness (Table 2). The mitochondrial genome of *A. flabelliformis* is remarkably similar to other Florideophyceae species in terms of gene content, gene sequences, gene organization, and AT composition [32,33,34,35,36,37,38].

Overall comparisons revealed that *A. flabelliformis* shares greater sequence similarity with *G. griffithsiae* than with *Mastocarpus* species, reflecting a closer evolutionary relationship within the Phyllophoraceae family. The presence of conserved synteny and shared gene content among these species indicates a high level of mitochondrial genome conservation, particularly in genes associated with core metabolic functions. The identified PCGs primarily encode components of the oxidative phosphorylation pathway—such as subunits of NADH dehydrogenase, cytochrome oxidase, and ATP synthase—highlighting their essential roles in mitochondrial energy production and strong functional constraints across lineages. Additionally, the presence of a complete set of tRNAs supports efficient mitochondrial translation. Despite these conserved features, differences observed in intergenic regions and specific ORFs suggest lineage-specific divergence, which may reflect adaptive responses to distinct ecological niches or environmental stressors encountered by *A. flabelliformis*.

### 3.2. Protein-Coding Gene Features

The *A. flabelliformis* mitochondrial genome consists of 24 PCGs (17,733 bp) comprised 68.22% of the total mitochondrial genome. *nad5* and *atp9* are the longest and shortest gene with 2004 bp and 231 bp in length, respectively (Table 1). The nucleotide composition and base skews value of PCGs are given in Table 3. The 24 PCGs in *A. flabelliformis* had an AT content of 71.8%; the values ranged from 66.4% for *cox1* to 78.4% for *tatC*. Additionally, there were five PCGs with positive AT-skew and fourteen PCGs with positive GC-skew, indicating base skews. Base skews result from strand-specific mutation rates, and replication-transcription biases which cause nucleotide composition variation [39]. The number of PCGs in the mitochondrial genomes of Phyllophoraceae species ranges from 23 to 25. Notably, *M*. *cristatus*, *M*. *latissimus*, and *M*. *papillatus* possess an additional hypothetical PCG, while *M*. *cristatus* and *M*. *stellatus* show a loss of the *rpl20* gene in their mitochondrial genome (Table 2 and Table 4). All PCGs of *A*. *flabelliformis* are identical; however, 14 PCGs vary in length among four *Mastocarpus* species and the *G*. *griffithsiae* (Table 4). The variation in PCGs lengths and base skews in red algal mitochondrial genomes is influenced by strand asymmetry, environmental adaptation, and evolutionary streamlining [40,41,42]. Genome reduction and selective pressure for compact mitochondrial genomes also resulted in differences in PCG lengths across red algal species [40,43,44]. The start codon for all PCGs in the *A*. *flabelliformis* mitochondrial genome was identified as ATG (Table 4). However, comparisons within the Phyllophoraceae family revealed variations, with GTG serving as the start codon for the *sdh4* gene in *M*. *cristatus*. Additionally, the *tatC* gene utilized both ATA and GTG, while the *atp6* gene initiated with ATT, ATC, and ATA start codons. In all PCGs, the stop codon TAA was utilized, with the exception of the TAG codon identified in the *cox1* gene of *A*. *flabelliformis*. Comparison with other species in the family Phyllophoraceae reveals the use of distinct stop codons in eight genes: TAA in the *cox1* gene and TAG in *atp4*, *cob*, *nad6*, *sdh2*, *rps11*, *nad4*, and *rpl20*. The overall sequencing, genomic structure, and organization of the *A. flabelliformis* mitochondrial genome were to be highly similar to other mitochondrial genomes from the order Gigartinales of class Florideophyceae. Comparative analysis has revealed that the mitochondrial genomes of other evolutionarily distant red algae, such as those from the classes Cyanidiophyceae and Bangiophyceae, higher sequence dissimilarities and genome rearrangements than those of the class Florideophyceae [40].

The codon usage and RSCU analysis results of *A. flabelliformis* mitochondrial PCGs are shown in Table S4. The total length of 24 PCGs in *A. flabelliformis* is 17,733 bp, containing total of 5887 codons (excluding stop codons). The number of encoded codons varies from 1 to 556, with a total of 62 codons encoding 20 amino acids, excluding the stop codons (UAA, UAG). The UUA codon for leucine had a high frequency of occurrence (N = 472) and the highest RSCU value (3.74 In this study, unbiased leucine (CUU) and methionine (AUG) codons were observed, and 28 codons among 62 codons showed a preference with an RSCU value more than 1, indicating a higher priority for these codons [45]. Out of the studied codons, 32 had a low bias, shown by their RSCU scores being less than 1. These findings are consistent with the preponderance of codons in the Phyllophoraceae species (*G*. *griffithsiae*, *M*. *cristatus*, *M*. *latissimus*, *M*. *papillatus*, and *M*. *stellatus*) (Table S4). These variations in codon use patterns across mitochondrial PCGs among species represent the complex linkage of evolutionary events including natural selection, genomic restrictions, and mutation pressure, which over time shapes the genetic composition of organisms [40,41,42,43,44]. More research is required to better understand the codon use bias of mitochondrial genes in red algae.

### 3.3. Ribosomal and Transfer RNA Genes

The mitochondrial genome of *A. flabelliformis* has an RNA genes composition that is both common and unusual among Phyllophoraceae species. Three rRNA genes (*rnl*, *rns*, and *rrn5*) were identified in the mitochondrial genome of *A. flabelliformis*, aligning with the characteristics observed in Phyllophoraceae species (Table S5). The *rnl* and *rns* were identified on the H strand, while *rrn5* was located on the L strand. Notably, the sizes of the *rnl* and *rns* genes in *A. flabelliformis* are larger compared to those in other species (*G*. *griffithsiae* and *Mastocarpus* species). The variations in genes composition are due to evolutionary changes [46].

The mitochondrial genome of *A. flabelliformis* comprises 22 tRNA genes, totaling 1665 bp, which accounts for 6.41% of the whole genome, with individual tRNA sizes ranging from 72 to 88 bp (Table 1), reflecting the structural diversity of RNA molecules. The number of tRNA genes in Phyllophoraceae species mitochondrial genomes ranges from 22 to 23 (Table S6). Notably, *A. flabelliformis* mitochondrial genome lacks the *trnI* gene-a feature also observed in other Phyllophoraceae species-suggesting a shared evolutionary loss or functional compensation mechanism within the family. In addition, *A. flabelliformis* shares the absence of *trnS* (GCT) with *M. stellatus* and *G. griffithsiae*. Although these species do not form a monophyletic clade, their shared gene absence implies independent gene loss events, supporting the idea of convergent evolution affecting mitochondrial tRNA content within Phyllophoraceae. In spite of these absences, the mitochondrial genome of *A. flabelliformis* retains characteristics typical of Phyllophoraceae, such as two codons for both the *trnL* and *trnR* genes and two copies of *trnM* (CAT). These patterns of gene presence, absence, and duplication highlight the dynamic evolutionary processes shaping mitochondrial genomes in red algae. While gene content among Florideophyceae species tends to be conserved, lineage-specific evolutionary pressures contribute to observed differences [44,46,47,48]. Functionally, the loss of certain mitochondrial tRNAs may not hinder translation, as nuclear-encoded tRNAs can be imported into the mitochondria-a compensatory mechanism previously reported in red algae [49,50].

### 3.4. Phylogenetic Relationship Within Gigartinales

**Figure 2:**
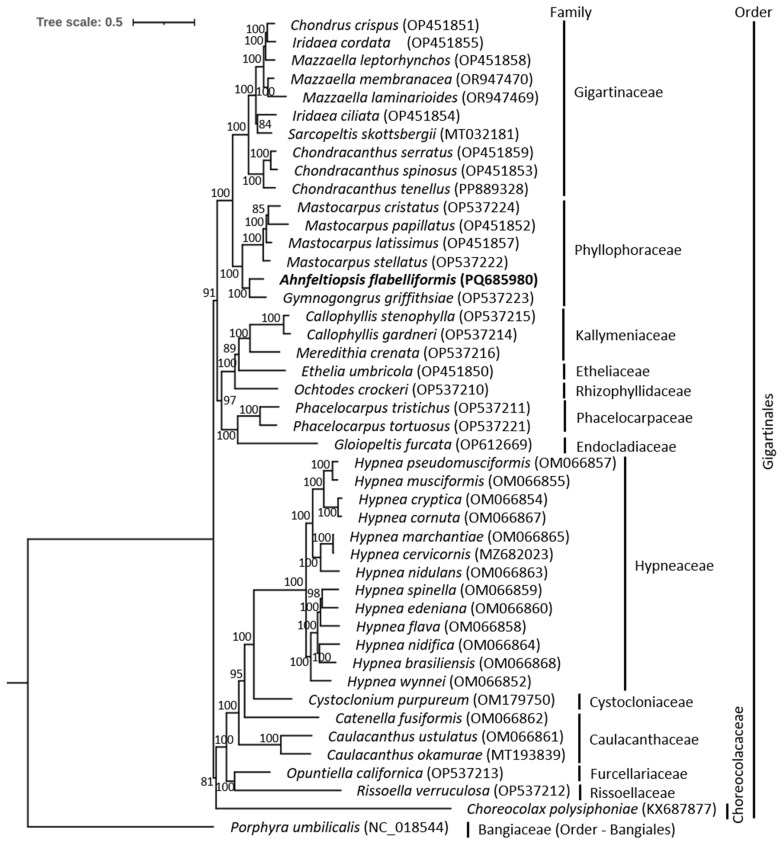
Maximum likelihood (ML) tree constructed using the complete mitochondrial genome sequences, effectively distinguishing *Ahnfeltiopsis flabelliformis* from other species of the Gigartinales order. The cladogram also offers perceptions into the evolutionary relations across diverse taxonomic levels within the Gigartinales order. ML bootstrap support values are annotated at each node, indicating the statistical support for individual branches in the topology.

The ML phylogenetic tree based on complete mitochondrial genome sequences of species within order Gigartinales shows the *A. flabelliformis* placed with *G. griffithsiae* and is closely related to *Mastocarpus* species within the Phyllophoraceae family (Figure 2). Strong bootstrap support values strengthen this grouping, indicating a well-supported evolutionary connection. The phylogenetic tree based on complete mitochondrial genome sequences of species within Phyllophoraceae also supports the placement of *A. flabelliformis* within the family Phyllophoraceae and closely related to *G. griffithsiae* and *Mastocarpus* species (Figure S2). The distinct separation of *A. flabelliformis* from Gigartinaceae species, suggests significant genetic divergence within the order Gigartinales. These findings are consistent with prior work that highlighted mitochondrial genome data as a reliable tool for determining evolutionary connections among red algae [40,44]. These results are consistent with previous studies and contribute to a better understanding of the evolutionary relationships within the Phyllophoraceae family. The newly sequenced mitochondrial genome of *A. flabelliformis* provides valuable phylogenetic relationships among Phyllophoraceae species, supporting taxonomic classifications. Moreover, comparative analysis reveals conserved and divergent regions that may reflect lineage-specific evolutionary pressures, contributing to a better understanding of mitochondrial genome evolution in Rhodophyta. Future studies incorporating additional *Ahnfeltiopsis* taxa could enhance our understanding of the genus’s evolutionary history and refine phylogenetic relationships within Phyllophoraceae.

## 4. Conclusions

This study determined the complete nucleotide sequence of the mitochondrial genome of *A. flabelliformis*. The comparative study revealed a high degree of similarity in the structure, organization, and gene content of the mitochondrial genome of *A. flabelliformis* compared to that of *G. griffithsiae*, its closest evolutionary relative with a fully sequenced mitochondrial genome. This similarity extends to other organisms within the family Phyllophoraceae, including *M. latissimus, M. papillatus, M. stellatus*, and *M. cristatus*. The phylogenetic relationship also indicates that *A. flabelliformis* is placed within the Phyllophoraceae family and closely related with *G. griffithsiae*. Further research into the molecular phylogeny of all species of the genus, as well as morphology and molecular biology, will be required to test and refine the above research.

## Figures and Tables

**Table 1:** List of annotated genes, including their boundaries, sizes, and intergenic nucleotides (IN), start and stop codons, anticodons and number of amino acids for *Ahnfeltiopsis flabelliformis*.

Gene	Position		Size (bp)	Coding Strand	IN	Codon		Anti-Codon	Amino Acids
	Start	End				Start	Stop		
*rnl*	1	2613	2613	H	0	-	-	-	-
*rps3*	2640	3332	693	H	26	ATG	TAA	-	230
*rpl16*	3344	3760	417	H	11	ATG	TAA	-	138
*trnD*	3765	3836	72	H	4	-	-	GTC	-
*cox1*	3879	5471	1593	H	42	ATG	TAA	-	530
*cox2*	5489	6250	762	H	17	ATG	TAA	-	253
*cox3*	6421	7239	819	H	170	ATG	TAA	-	272
*atp4*	7250	7798	549	H	10	ATG	TAA	-	182
*trnG*	7808	7881	74	H	9	-	-	TCC	-
*trnQ*	7884	7955	72	H	2	-	-	TTG	-
*trnL*	8062	8145	84	H	106	-	-	TAA	-
*cob*	8192	9334	1143	H	46	ATG	TAA	-	380
*trnL*	9394	9476	83	L	59	-	-	TAG	-
*nad6*	9481	10,098	618	L	4	ATG	TAA	-	205
*trnG*	10,123	10,195	73	L	24	-	-	GCC	-
*trnH*	10,208	10,282	75	H	12	-	-	GTG	-
*sdh2*	10,283	11,041	759	L	0	ATG	TAA	-	252
*sdh3*	11,046	11,447	402	L	4	ATG	TAA	-	133
*trnF*	11,470	11,541	72	L	22	-	-	GAA	-
*trnS*	11,561	11,644	84	L	19	-	-	TGA	-
*trnP*	11,655	11,727	73	L	10	-	-	TGG	-
*atp9*	11,753	11,983	231	L	25	ATG	TAA	-	76
*trnC*	12,027	12,098	72	L	43	-	-	GCA	-
*trnM*	12,105	12,187	83	L	6	-	-	CAT	-
*rps11*	12,190	12,546	357	L	2	ATG	TAA	-	118
*rrn5*	12,568	12,675	108	L	21	-	-	-	-
*nad3*	12,688	13,053	366	L	12	ATG	TAA	-	121
*nad1*	13,075	14,055	981	L	21	ATG	TAA	-	326
*nad2*	14,082	15,575	1494	L	26	ATG	TAA	-	497
*sdh4*	15,592	15,831	240	L	16	ATG	TAA	-	79
*nad4*	15,848	17,329	1482	L	16	ATG	TAA	-	493
*nad5*	17,910	19,913	2004	L	580	ATG	TAA	-	667
*atp8*	19,925	20,335	411	L	11	ATG	TAA	-	136
*atp6*	20,348	21,109	762	L	12	ATG	TAA	-	253
*trnW*	21,147	21,218	72	L	37	-	-	TCA	-
*trnA*	21,739	21,812	74	L	520	-	-	TGC	-
*trnR*	21,846	21,919	74	L	33	-	-	TCT	-
*trnY*	21,931	22,018	88	L	11	-	-	GTA	-
*trnN*	22,330	22,402	73	H	311	-	-	GTT	-
*trnV*	22,410	22,482	73	H	7	-	-	TAC	-
*trnR*	22,530	22,604	75	H	47	-	-	ACG	-
*trnK*	22,632	22,705	74	H	27	-	-	TTT	-
*tatC*	22,753	23,460	708	H	47	ATG	TAA	-	235
*rps12*	23,461	23,844	384	H	0	ATG	TAA	-	127
*trnE*	23,830	23,901	72	H	−16	-	-	TTC	-
*trnM*	23,908	23,980	73	H	6	-	-	CAT	-
*rpl20*	23,992	24,243	252	H	11	ATG	TAA	-	83
*rns*	24,276	25,648	1373	H	32	-	-	-	-
*nad4L*	25,687	25,992	306	H	38	ATG	TAA	-	101

**Table 2:** General features of the complete mitochondrial genome of Phyllophoraceae species (ORF* indicates unidentified reading frame).

Species (Accession No.)	Size (bp)	Nucleotide Composition (%)						AT-Skew	GC-Skew	Number of Genes			
		A	T	G	C	A + T	G + C			PCG	tRNA	rRNA	ORF*
*Ahnfeltiopsis flabelliformis* (PQ685980)	25,992	37.4	33.9	14.7	14.0	71.3	28.7	0.049	0.023	24	22	3	0
*Gymnogongrus griffithsiae* (OP537223)	25,812	37.0	33.6	15.2	14.2	70.6	29.4	0.048	0.035	24	22	3	0
*Mastocarpus cristatus* (OP537224)	25,838	35.5	31.8	16.7	16.0	67.3	32.7	0.054	0.023	24	23	3	1
*Mastocarpus latissimus* (OP451857)	26,208	36.2	32.2	16.2	15.5	68.4	31.6	0.059	0.023	25	23	3	1
*Mastocarpus papillatus* (OP451852)	26,132	34.3	30.6	18.1	17.0	64.9	35.1	0.058	0.023	25	23	3	1
*Mastocarpus stellatus* (OP537222)	25,826	36.4	32.5	15.9	15.2	68.9	31.1	0.057	0.022	23	22	3	0

**Table 3:** Nucleotide composition and skewness of PCGs in the mitochondrial genome of *Ahnfeltiopsis flabelliformis*.

PCG	Length (bp)	Nucleotide Composition (%)						AT-Skewness	GC-Skewness
		A	T	G	C	A + T	G + C		
*rps3*	693	37.2	36.9	12.3	13.6	74.2	25.8	0.004	−0.050
*rpl16*	417	41.2	31.4	13.9	13.4	72.7	27.3	0.135	0.018
*cox1*	1593	26.9	39.5	17.0	16.6	66.4	33.6	−0.189	0.013
*cox2*	762	31.1	36.5	17.5	15.0	67.6	32.4	−0.080	0.077
*cox3*	819	25.8	42.9	17.2	14.2	68.6	31.4	−0.249	0.097
*atp4*	549	37.5	39.2	9.3	14.0	76.7	23.3	−0.021	−0.203
*cob*	1143	27.0	42.6	16.1	14.3	69.6	30.4	−0.224	0.061
*nad6*	618	29.1	42.9	15.0	12.9	72.0	28.0	−0.191	0.075
*sdh2*	759	35.7	36.2	13.7	14.4	71.9	28.1	−0.007	−0.023
*sdh3*	402	28.6	47.5	8.2	15.7	76.1	23.9	−0.248	−0.313
*atp9*	231	26.0	40.7	20.3	13.0	66.7	33.3	−0.221	0.221
*rps11*	357	42.6	32.8	12.0	12.6	75.4	24.6	0.130	−0.023
*nad3*	366	30.3	45.9	13.7	10.1	76.2	23.8	−0.204	0.149
*nad1*	981	28.7	40.8	16.5	14.0	69.5	30.5	−0.173	0.084
*nad2*	1494	28.1	46.0	13.3	12.6	74.1	25.9	−0.241	0.028
*sdh4*	240	35.0	43.3	10.4	11.3	78.3	21.7	−0.106	−0.038
*nad4*	1482	27.6	44.5	14.0	13.9	72.1	27.9	−0.234	0.005
*nad5*	2004	26.4	44.0	16.1	13.5	70.5	29.5	−0.249	0.088
*atp8*	411	38.4	38.7	8.5	14.4	77.1	22.9	−0.003	−0.255
*atp6*	762	28.7	43.8	13.0	14.4	72.6	27.4	−0.208	−0.053
*tatC*	708	28.4	50.0	9.3	12.3	78.4	21.6	−0.276	−0.137
*rps12*	384	38.3	29.9	17.2	14.6	68.2	31.8	0.122	0.082
*rpl20*	252	42.9	35.3	9.9	11.9	78.2	21.8	0.096	−0.091
*nad4L*	306	32.7	41.2	14.4	11.8	73.9	26.1	−0.115	0.100
Total	17,733	30.3	41.5	14.3	13.08	71.8	28.2	-	-

**Table 4:** Characteristics of the mitochondrial PCGs in six Phyllophoraceae species.

Genes	Length (bp)	Amino Acids	Start Codon	Stop Codon	Coding Strand
*rps3*	693	230	ATG	TAA	H
*rpl16*	417 ^a,d^/435 ^b^/414 ^c^/411 ^e^/420 ^f^	138 ^a,d^/144 ^b^/137 ^c^/136 ^e^/139 ^f^	ATG	TAA	H
*cox1*	1593 ^a^/1596 ^b^/1608 ^c,d,e,f^	530 ^a^/531 ^b^/535 ^c,d,e,f^	ATG	TAG ^a,b^/TAA ^c,d,e,f^	H
*cox2*	762 ^a^/771 ^b,c,d,e,f^	253 ^a^/256 ^b,c,d,e,f^	ATG	TAA	H
*cox3*	819	272	ATG	TAA	H
*atp4*	549 ^a,b^/552 ^c,d,e,f^	182 ^a,b^/183 ^c,d,e,f^	ATG	TAA ^a,b,c,e,f^/TAG ^d^	H
*cob*	1143 ^a,b^/1146 ^c,d,e^/1152 ^f^	380 ^a,b^/381 ^c,d,e^/383 ^f^	ATG	TAA ^a,b,c,e,f^/TAG ^d^	H
*nad6*	618 ^a,c,e^/609 ^b^/615 ^d,f^	205 ^a,c,e^/202 ^b^/204 ^d,f^	ATG	TAA ^a,c,d,e,f^/TAG ^b^	L
*sdh2*	759 ^a^/762 ^b^/753 ^c,d,e,f^	252 ^a^/253 ^b^/250 ^c,d,e,f^	ATG	TAA ^a,b,c^/TAG ^d,e,f^	L
*sdh3*	402 ^a,b,d,f^/447 ^c^/396 ^e^	133 ^a,b,d,f^/148 ^c^/131 ^e^	ATG	TAA	L
*atp9*	231	76	ATG	TAA	L
*rps11*	357 ^a,b^/360 ^c,d,e,f^	118 ^a,b^/119 ^c,d,e,f^	ATG	TAA ^a,b,c,d,f^/TAG ^e^	L
*nad3*	366	121	ATG	TAA	L
*nad1*	981	326	ATG	TAA	L
*nad2*	1494	497	ATG	TAA	L
*sdh4*	240	79	ATG ^a,b,d,e,f^/GTG ^c^	TAA	L
*nad4*	1482 ^a,c^/1479 ^b^/1473 ^d,e,f^	493 ^a,c^/492 ^b^/490 ^d,e,f^	ATG	TAA ^a,b,c,f^/TAG ^d,e^	L
*nad5*	2004	667	ATG	TAA	L
*atp8*	411 ^a^/414 ^b,c,d,e,f^	136 ^a^/137 ^b,c,d,e,f^	ATG	TAA	L
*atp6*	762 ^a^/771 ^b,c,d,e,f^	253 ^a^/256 ^b,c,d,e,f^	ATG ^a^/ATT ^b^/ATC ^c,d,e^/ATA ^f^	TAA	L
*tatC*	708 ^a^/783 ^b^/738 ^c,d,f^/543 ^e^	235 ^a^/260 ^b^/245 ^c,d,f^/180 ^e^	ATG ^a^/ATA ^b,c,d,f^/GTG ^e^	TAA	H
*rps12*	384	127	ATG	TAA	H
rpl20 *	252 ^a,b,d^/264 ^e^	83 ^a,b,d^/87 ^e^	ATG ^a,b,d,e^	TAA ^a,b,d^/TAG ^e^	H
*nad4L*	306	101	ATG	TAA	H

^a^ *A. flabelliformis* (PQ685980); ^b^ *G. griffithsiae* (OP537223); ^c^ *M. cristatus* (OP537224); ^d^ *M. latissimus* (OP451857); ^e^ *M. papillatus* (OP451852); ^f^ *M. stellatus* (OP537222); * gene *rpl20* absent in *M. cristatus*, and *M. stellatus*.

## Data Availability

Data associated with this study has been deposited at NCBI under the accession number PQ685980 (https://www.ncbi.nlm.nih.gov/nuccore/PQ685980). All data generated or analyzed during this study are included in this article and its Supplementary Information Files.

## Supplementary Materials

The following supporting information can be downloaded at: https://www.mdpi.com/article/10.3390/biology14060638/s1, Figure S1: A specimen image of *Ahnfeltiopsis flabelliformis* (family Phyllophoraceae) collected from the coast of Busan, South Korea. This marine macroalga is 5 to 8 cm long, cartilaginous, and flat-thallus, with dichotomous or irregular branching, fan-shaped (flabelliform) with deep red to reddish-brown color and lighter-colored tips or shoots; Figure S2: Maximum Likelihood (ML) topology constructed using the complete mitochondrial genome sequences of *A. flabelliformis* (in this study) and other five species belongs to the family Phyllophoraceae and *Porphyra umblicalis* as an outgroup member. ML bootstrap support values are annotated at each node, indicating the statistical support for individual branches in the topology; Table S1: List of the species from Gigartinales order used in this study; Table S2: Summary of *Ahnfeltiopsis flabelliformis* mitochondrial genome data produced/stats during de novo assembly analysis in Illumina platform using NOVOPlasty v4.2.1 assembly method; Table S3: Annotation of mitochondrial functional genes of *Ahnfeltiopsis flabelliformis*; Table S4: Relative Synonymous Codon Usage (RSCU) values of complete protein-coding genes in the mitochondrial genome of Phyllophoraceae species; Table S5: Mitochondrial rRNA in Phyllophoraceae species; Table S6: Mitochondrial tRNA in Phyllophoraceae species.
