# Supplementary material for: Characterization of the Complete Mitochondrial Genome of the Red Alga Ahnfeltiopsis flabelliformis (Rhodophyta, Gigartinales, Phyllophoraceae) and Its Phylogenetic Analysis
Source: Biology (Basel). 2025 May 30;14(6):638. doi: 10.3390/biology14060638 (PMC12189398; doi:10.3390/biology14060638)
Supplement: Supplementary file 1 [file biology-14-00638-s001.zip › biology-3574362-supplementary.pdf]

Supplementary Materials

Characterization of the Complete Mitochondrial Genome of the Red Alga *Ahnfeltiopsis flabelliformis* (Rhodophyta, Gigartinales, Phyllophoraceae) and Its Phylogenetic Analysis

Figures:

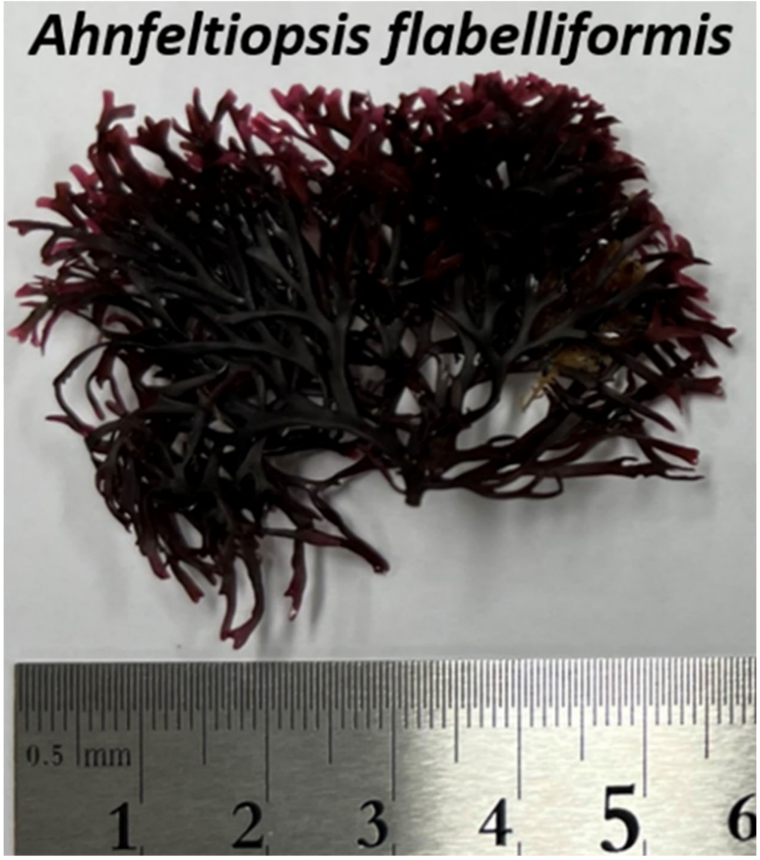

Figure S1. A specimen image of *Ahnfeltiopsis flabelliformis* (family Phyllophoraceae) collected from the coast of Busan, South Korea. This marine macroalga is 5 to 8 cm long, cartilaginous, and flat-thallus, with dichotomous or irregular branching, fan-shaped (flabelliform) with deep red to reddish-brown color and lighter-colored tips or shoots.

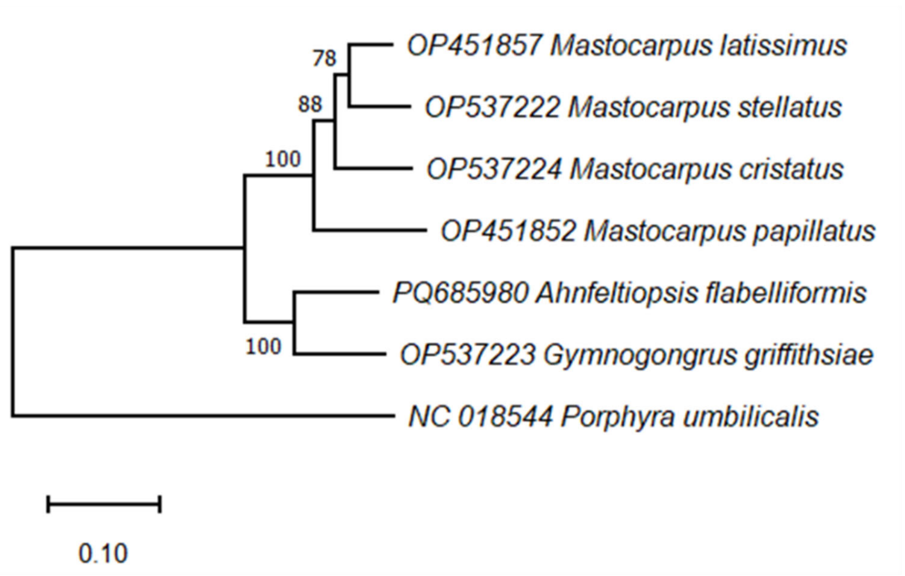

Figure S2. Maximum Likelihood (ML) topology constructed using the complete mitochondrial genome sequences of *A. flabelliformis* (in this study) and other five species belongs to the family Phyllophoraceae and *Porphyra umblicalis* as an outgroup member. ML bootstrap support values are annotated at each node, indicating the statistical support for individual branches in the topology.

Tables:

Table S1. List of the species from Gigartinales order used in this study.

Family	Species	Accession No.	Length (bp)
Hypneaceae	*Hypnea cryptica*	OM066854	25,190
	Hypnea musciformis	OM066855	25,183
	Hypnea brasiliensis	OM066868	25,170
	Hypnea spinella	OM066859	25,165
	Hypnea cornuta	OM066867	25,129
	Hypnea wynnei	OM066852	25,126
	Hypnea nidulans	OM066863	25,110
	Hypnea pseudomusciformis	OM066857	25,093
	Hypnea flava	OM066858	25,085
	Hypnea nidifica	OM066864	25,082
	Hypnea edeniana	OM066860	25,073
	Hypnea marchantiae	OM066865	25,061
	Hypnea cervicornis	MZ682023	25,060
Gigartinaceae	*Iridaea ciliata*	OP451854	26,466
	Iridaea cordata	OP451855	25,703
	Chondrus crispus	OP451851	25,694
	Chondracanthus spinosus	OP451853	26,057
	Chondracanthus serratus	OP451859	25,943
	Chondracanthus tenellus	PP889328	25,928
	Sarcopeltis skottsbergii	MT032181	25,908
	Mazzaella membranacea	OR947470	28,716
	Mazzaella laminarioides	OR947469	25,695
	Mazzaella leptorhynchos	OP451858	25,684
Phyllophoraceae	*Ahnfeltiopsis flabelliformis*	PQ685980	25,992
	Mastocarpus latissimus	OP451857	26,208
	Mastocarpus papillatus	OP451852	26,132
	Mastocarpus stellatus	OP537222	25,826
	Mastocarpus cristatus	OP537224	25,838
	Gymnogongrus griffithsiae	OP537223	25,812
Kallymeniaceae	*Callophyllis gardneri*	OP537214	32,648
	Callophyllis stenophylla	OP537215	29,402
	Meredithia crenata	OP537216	31,338
Caulacanthaceae	*Caulacanthus okamurae*	MT193839	25,995
	Caulacanthus ustulatus	OM066861	25,810
	Catenella fusiformis	OM066862	25,174
Phacelocarpaceae	*Phacelocarpus tristichus*	OP537211	28,911
	Phacelocarpus tortuosus	OP537221	28,639
Endocladiaceae	*Gloiopeltis furcata*	OP612669	26,600
Rissoellaceae	*Rissoella verruculosa*	OP537212	26,302
Rhizophyllidaceae	*Ochtodes crockeri*	OP537210	28,903

Etheliaceae	*Ethelia umbricola*	OP451850	25,658
Choreocolacaceae	*Choreocolax polysiphoniae*	KX687877	25,357
Cystocloniaceae	*Cystoclonium purpureum*	OM179750	24,260
Furcellariaceae	*Opuntiella californica*	OP537213	26,717
Bangiaceae (Out group member)	*Porphyra umbilicalis*	NC_018544	29,123

Table S2. Summary of *Ahnfeltiopsis flabelliformis* mitochondrial genome data produced/ stats during de novo assembly analysis in Illumina platform using NOVOPlasty v4.2.1 assembly method.

Library	Total read bases	Total reads	GC (%)	Q20 (%)	Q30 (%)	Mapped reads	Coverage (%)	Depth	Insert size (std.)
Raw data stats	2,188,047,984	14,490,384	46.38	96.28	91.92	-	-	-	-
Filtered data stats	1,828,882,139	12,185,016	46.03	99.19	96.98	-	-	-	-
Self-mapping stats	-	12,185,016	-	-	-	25,933	100.00	124.75	340.58 (124.56)

(Coverage %: The percentage of mapped sites (>= 1x). Depth: Average mapping depth. Insert size (std.): The length between adapters and the standard deviation of predicted length.)

Table S3. Annotation of mitochondrial functional genes of *Ahnfeltiopsis flabelliformis*

Type	Group of Gene		Name of Gene
RNAs	rRNA	The large subunit of a ribosome	*rnl*
		The small subunit of a ribosome	*rrn5*, *rns*
	tRNA	Transfer RNA genes	*trnA*, *trnC*, *trnD*, *trnE*, *trnF*, *trnG*1, *trnG2*, *trnH*, *trnK*, *trnL*1, *trnL*2, *trnM*1, *trnM*2, *trnN*, *trnP*, *trnQ*, *trnR*1, *trnR*2, *trnS*, *trnV*, *trnW*, *trnY*
PCGs	Respiratory chain subunits	NADH dehydrogenase subunits (complex I)	*nad1*, *nad2*, *nad3*, *nad4*, *nad5*, *nad6*, *nad4L*
		Succinate dehydrogenase subunit (complex II)	*sdh2*, *sdh3*, *sdh4*
		Apocytochrome b (complex III)	*cob*
		Cytochrome c oxidase subunit (complex IV)	*cox1*, *cox2*, *cox3*
	ATP synthase subunits (complex V)		*atp6*, *atp8*, *atp9*, *atp4*
	Ribosomal proteins	Small subunit (SSU)	*rps3*, *rps11*, *rps12*
		Large subunit (LSU)	*rpl16*, *rpl20*
	Independent protein translocase		*tatC*

Table S4. Relative Synonymous Codon Usage (RSCU) values of complete protein-coding genes in the mitochondrial genome of Phyllophoraceae species.

Codon	*A. flabelliformis*		*G. griffithsiae*		*M. cristatus*		*M. latissimus*		*M. papillatus*		*M. stellatus*	
	**Count**	**RSCU**	**Count**	**RSCU**	**Count**	**RSCU**	**Count**	**RSCU**	**Count**	**RSCU**	**Count**	**RSCU**
UUU(F)	472	1.71	452	1.65	384	1.42	406	1.44	344	1.31	408	1.51
UUC(F)	79	0.29	96	0.35	158	0.58	156	0.56	181	0.69	134	0.49
UUA(L)	556	3.74	537	3.62	441	2.87	476	3.09	361	2.33	469	3.20
UUG(L)	77	0.52	83	0.56	126	0.82	117	0.76	139	0.90	104	0.71
CUU(L)	148	1.00	149	1.00	149	0.97	149	0.97	159	1.03	130	0.89
CUC(L)	15	0.10	12	0.08	28	0.18	31	0.20	51	0.33	34	0.23
CUA(L)	85	0.57	95	0.64	143	0.93	125	0.81	172	1.11	125	0.85
CUG(L)	10	0.07	14	0.09	35	0.23	26	0.17	46	0.30	18	0.12
AUU(I)	449	2.10	414	1.98	339	1.73	377	1.82	303	1.69	379	1.87
AUC(I)	59	0.28	71	0.34	125	0.64	101	0.49	123	0.69	89	0.44
AUA(I)	133	0.62	141	0.68	125	0.64	142	0.69	112	0.62	140	0.69
AUG(M)	158	1.00	161	1.00	163	1.00	166	1.00	160	1.00	160	1.00
GUU(V)	197	2.28	194	2.19	177	1.90	180	2.07	187	1.87	182	2.10
GUC(V)	20	0.23	23	0.26	51	0.55	38	0.44	67	0.67	42	0.48
GUA(V)	111	1.29	120	1.35	116	1.24	105	1.21	113	1.13	99	1.14
GUG(V)	17	0.20	18	0.20	29	0.31	25	0.29	34	0.34	24	0.28
UCU(S)	147	1.69	153	1.75	166	1.73	177	1.85	142	1.51	156	1.71
UCC(S)	14	0.16	23	0.26	41	0.43	31	0.32	58	0.62	34	0.37
UCA(S)	190	2.18	155	1.77	150	1.57	154	1.61	139	1.48	151	1.66
UCG(S)	33	0.38	49	0.56	56	0.58	60	0.63	77	0.82	60	0.66
CCU(P)	93	1.90	92	1.83	97	1.82	91	1.78	73	1.39	86	1.76
CCC(P)	15	0.31	16	0.32	23	0.43	24	0.47	41	0.78	21	0.43
CCA(P)	76	1.55	78	1.55	63	1.18	61	1.20	61	1.16	70	1.43
CCG(P)	12	0.24	15	0.30	30	0.56	28	0.55	35	0.67	19	0.39
ACU(T)	137	1.85	146	1.83	141	1.71	166	2.01	143	1.62	149	1.94
ACC(T)	35	0.47	38	0.48	61	0.74	35	0.42	67	0.76	37	0.48
ACA(T)	99	1.34	114	1.43	88	1.07	93	1.13	82	0.93	91	1.18
ACG(T)	25	0.34	22	0.28	40	0.48	36	0.44	61	0.69	31	0.40
GCU(A)	137	1.96	136	2.00	119	1.65	120	1.66	105	1.46	121	1.69
GCC(A)	17	0.24	23	0.34	30	0.42	37	0.51	45	0.63	32	0.45
GCA(A)	115	1.65	92	1.35	109	1.51	99	1.37	88	1.22	93	1.30
GCG(A)	10	0.14	21	0.31	31	0.43	34	0.47	50	0.69	41	0.57
UAU(Y)	188	1.48	199	1.52	157	1.23	162	1.24	122	0.98	170	1.35
UAC(Y)	66	0.52	62	0.48	98	0.77	100	0.76	127	1.02	82	0.65
UAA(*)	0	0	0	0	0	0	0	0	0	0	0	0
UAG(*)	0	0	0	0	0	0	0	0	0	0	0	0
CAU(H)	94	1.68	99	1.62	85	1.35	75	1.23	66	1.07	84	1.38
CAC(H)	18	0.32	23	0.38	41	0.65	47	0.77	57	0.93	38	0.62

Codon	*A. flabelliformis*		*G. griffithsiae*		*M. cristatus*		*M. latissimus*		*M. papillatus*		*M. stellatus*	
	**Count**	**RSCU**	**Count**	**RSCU**	**Count**	**RSCU**	**Count**	**RSCU**	**Count**	**RSCU**	**Count**	**RSCU**
CAA(Q)	134	1.71	125	1.61	127	1.51	139	1.62	134	1.65	125	1.61
CAG(Q)	23	0.29	30	0.39	41	0.49	33	0.38	29	0.35	30	0.39
AAU(N)	206	1.54	202	1.56	165	1.31	171	1.31	142	1.13	175	1.43
AAC(N)	61	0.46	57	0.44	87	0.69	91	0.69	110	0.87	70	0.57
AAA(K)	216	1.66	230	1.72	214	1.67	236	1.72	219	1.58	209	1.69
AAG(K)	44	0.34	37	0.28	42	0.33	39	0.28	59	0.42	39	0.31
GAU(D)	104	1.68	107	1.66	87	1.35	100	1.49	79	1.22	98	1.62
GAC(D)	20	0.32	22	0.34	42	0.65	34	0.51	51	0.78	23	0.38
GAA(E)	130	1.59	145	1.74	128	1.50	132	1.55	127	1.55	121	1.48
GAG(E)	34	0.41	22	0.26	43	0.50	38	0.45	37	0.445	42	0.52
UGU(C)	64	1.71	51	1.36	45	1.20	46	1.28	44	1.17	50	1.43
UGC(C)	11	0.29	24	0.64	30	0.80	26	0.72	31	0.83	20	0.57
UGA(W)	106	1.72	103	1.72	97	1.60	100	1.64	96	1.52	103	1.70
UGG(W)	17	0.28	17	0.28	24	0.40	22	0.36	30	0.48	18	0.30
CGU(R)	59	2.34	52	2.09	47	1.94	49	1.92	47	1.76	47	1.94
CGC(R)	6	0.24	7	0.28	13	0.54	13	0.51	21	0.79	13	0.54
CGA(R)	32	1.27	31	1.25	25	1.03	27	1.06	29	1.09	22	0.91
CGG(R)	1	0.04	6	0.24	6	0.25	2	0.08	3	0.11	2	0.08
AGU(S)	112	1.29	110	1.26	112	1.17	117	1.23	91	0.97	91	1.00
AGC(S)	26	0.30	34	0.39	50	0.52	34	0.36	56	0.60	54	0.59
AGA(R)	51	2.03	46	1.85	46	1.90	57	2.24	48	1.80	57	2.36
AGG(R)	2	0.08	7	0.28	8	0.33	5	0.20	12	0.45	4	0.17
GGU(G)	129	1.61	144	1.79	137	1.75	149	1.91	128	1.59	136	1.75
GGC(G)	16	0.20	15	0.19	36	0.46	17	0.22	50	0.62	23	0.30
GGA(G)	136	1.69	133	1.65	106	1.35	112	1.47	108	1.34	120	1.55
GGG(G)	40	0.50	30	0.37	35	0.45	31	0.40	36	0.45	31	0.40
Total	5887	-	5923	-	6008	-	6073	-	6009	-	5826	-

Note: * = the termination codon, A = Ala, F = Phe, C = Cys, D = Asp, N = Asn, E = Glu, Q = Gln, G = Gly, H = His, L = Leu, I = Ile, K = Lys, M = Met, P = Pro, R = Arg, S = Ser, T = Thr, V = Val, W = Trp, Y = Tyr

Table S5. Mitochondrial rRNA in Phyllophoraceae species.

**rRNA**	***A. flabelliformis***	***G. griffithsiae***	***M. cristatus***	***M. latissimus***	***M. papillatus***	***M. stellatus***
rrn5	108 bp	109 bp	108 bp	108 bp	29 bp	110 bp
rns	1,373 bp	1,367 bp	1,365 bp	1,365 bp	1,364 bp	1,363 bp

rRNA	*A. flabelliformis*	*G. griffithsiae*	*M. cristatus*	*M. latissimus*	*M. papillatus*	*M. stellatus*
rnl	2,613 bp	2,588 bp	2,592 bp	2,581 bp	2,592 bp	2,594 bp

Table S6. Mitochondrial tRNA in Phyllophoraceae species.

tRNA	*A. flabelliformis*	*G. griffithsiae*	*M. cristatus*	*M. latissimus*	*M. papillatus*	*M. stellatus*
trnA (TGC)	1	1	1	1	1	1
trnC (GCA)	1	1	1	1	1	1
trnD (GTC)	1	1	1	1	1	1
trnE (TTC)	1	1	1	1	1	1
trnF (GAA)	1	1	1	1	1	1
trnG (TCC)	1	1	0	0	1	0
trnG (GCC)	1	1	2	2	1	2
trnH (GTG)	1	1	1	1	1	1
trnI (GAT)	0	0	0	0	0	0
trnK (TTT)	1	1	1	1	1	1
trnL (TAA)	1	1	1	1	1	1
trnL (TAG)	1	1	1	1	1	1
trnM (CAT)	2	2	2	2	2	2
trnN (GTT)	1	1	1	1	1	1
trnP (TGG)	1	1	1	1	1	1
trnQ (TTG)	1	1	1	1	1	1
trnR (ACG)	1	1	1	1	1	1
trnR (TCT)	1	1	1	1	1	1
trnS (GCT)	0	0	1	1	1	0
trnS (TGA)	1	1	1	1	1	2
trnV (TAC)	1	1	1	1	1	1
trnW (TCA)	1	1	1	1	1	1
trnY (GTA)	1	1	1	1	1	0
Total tRNA	22	22	23	23	23	22
